# In-hospital mortality during the wild-type, alpha, delta, and omicron SARS-CoV-2 waves: a multinational cohort study in the EuCARE project

**DOI:** 10.1016/j.lanepe.2024.100855

**Published:** 2024-02-02

**Authors:** Pontus Hedberg, Milosz Parczewski, Karol Serwin, Giulia Marchetti, Francesca Bai, Björn-Erik Ole Jensen, Joana P.V. Pereira, Francis Drobniewski, Henrik Reschreiter, Daniel Naumovas, Francesca Ceccherini-Silberstein, Gibran Horemheb Rubio Quintanares, Matilu Mwau, Cristina Toscano, Florian König, Nico Pfeifer, Maurizio Zazzi, Iuri Fanti, Francesca Incardona, Alessandro Cozzi-Lepri, Anders Sönnerborg, Pontus Nauclér

**Affiliations:** a Department of Medicine Huddinge, Karolinska Institutet, Stockholm, Sweden; b Department of Tropical Infectious Diseases and Immune Deficiency, Pomeranian Medical University in Szczecin, Szczecin, Poland; c Department of Health Sciences, Clinic of Infectious Diseases, ASST Santi Paolo E Carlo, University of Milan, Milan, Italy; d Department of Gastroenterology, Hepatology and Infectious Diseases, Medical Faculty and University Hospital Duesseldorf, Heinrich Heine University, Duesseldorf, Germany; e Department of Infectious Disease, Imperial College, London, W12 0NN, UK; f University Hospital Dorset, Poole Hospital, Poole, Dorset, UK; g Vilnius Santaros Klinikos Biobank, Vilnius University Hospital Santaros Klinikos, Vilnius, Lithuania; h Department of Experimental Medicine, University of Rome Tor Vergata, Rome, Italy; i Virus Security Department, Paul Ehrlich Institute, Langen, Germany; j Infectious Diseases Department, Instituto Nacional de Ciencias Médicas y Nutrición Salvador Zubirán, Mexico; k Center for Infectious and Parasitic Diseases Control Research, Kenya Medical Research Institute, Busia, Kenya; l Microbiology Laboratory, Centro Hospitalar de Lisboa Ocidental, Lisboa, Portugal; m Institute for Bioinformatics and Medical Informatics and Medical Informatics (IBMI), University of Tübingen, Tübingen, Germany; n Methods in Medical Informatics Department of Computer Science, University of Tübingen, Tübingen, Germany; o Department of Medical Biotechnologies, University of Siena, Italy; p EuResist Network GEIE, Via Guido Guinizelli, 98/100, 00152, Roma, Italy; q InformaPRO s.r.l., Via Guido Guinizelli, 98/100, 00152, Roma, Italy; r Centre for Clinical Research, Epidemiology, Modelling and Evaluation (CREME) Institute for Global Health UCL, Rowland Hill St, London, NW3 2PF, UK; s Department of Infectious Diseases, Karolinska University Hospital, Stockholm, Sweden; t Division of Infectious Diseases, Department of Medicine, Solna, Karolinska Institutet, Stockholm, Sweden

**Keywords:** SARS-CoV-2, COVID-19, Variants of concern, In-hospital mortality

## Abstract

**Background:**

Investigating outcomes of hospitalised COVID-19 patients throughout the pandemic is crucial to understand the impact of different SARS-CoV-2 variants. We compared 28-day in-hospital mortality of Wild-type, Alpha, Delta, and Omicron variant infections. Whether the difference in risk by variant varied by age was also evaluated.

**Methods:**

We conducted a cohort study including patients ≥18 years, hospitalised between 2020 and 02-01 and 2022-10-15 with a SARS-CoV-2 positive test, from nine countries. Variant was classified based on sequenced viruses or from national public metadata. Mortality was compared using the cumulative incidence function and subdistribution hazard ratios (SHR) adjusted for age, sex, calendar time, and comorbidities. Results were shown age-stratified due to effect measure modification (*P* < 0.0001 for interaction between age and variant).

**Findings:**

We included 38,585 participants: 19,763 Wild-type, 6387 Alpha, 3640 Delta, and 8795 Omicron. The cumulative incidence of mortality decreased throughout the study period. Among participants ≥70 years, the adjusted SHR (95% confidence interval) for Delta vs. Omicron was 1.66 (1.29–2.13). This estimate was 1.66 (1.17–2.36) for Alpha vs. Omicron, and 1.34 (0.92–1.95) for Wild-type vs. Omicron. These were 1.21 (0.81–1.82), 1.21 (0.68–2.17), and 0.98 (0.53–1.82) among unvaccinated participants. When comparing Omicron sublineages, the aSHR for BA.1 was 1.92 (1.43–2.58) compared to BA.2 and 1.52 (1.11–2.08) compared to BA.5.

**Interpretation:**

The herein observed decrease in in-hospital mortality seems to reflect a combined effect of immunity from vaccinations and previous infections, although differences in virulence between SARS-CoV-2 variants may also have contributed.

**Funding:**

European Union’s Horizon Europe Research and Innovation Programme.

**Research in context**

**Evidence before this study**

We aimed to identify peer-reviewed studies comparing in-hospital mortality of adult patients hospitalised with COVID-19 during different SARS-CoV-2 variant waves. This was done using the following search query in the PubMed database from database inception to 9 October 2023 (date of the latest literature review): (“SARS-CoV-2” OR “severe acute respiratory syndrome coronavirus 2” OR “COVID-19” OR “Coronavirus disease 2019”) AND (“In-hospital mortality” OR “In hospital mortality” OR “In-hospital death” OR “In hospital death”). The search yielded 3664 studies. Furthermore, other studies elsewhere identified by the authors were also considered. Overall, most original research articles were confined to one centre, one region, or one country, and indicated in-hospital mortality to decrease throughout the pandemic, in particular since the emergence of the Omicron variant. A meta-analysis of 7 studies of hospitalised patients found Delta to be associated with an increased mortality risk compared with Omicron (Odds Ratio 1.44, 95% confidence interval [CI] 1.22–1.71). A study from the US, comparing the in-hospital mortality during the Delta (July to October 2021), early Omicron (January to March 2022), and later Omicron (April to June 2022) periods found the adjusted mortality risk difference to be −5.3 (95% CI −5.5 to −5.0) for early Omicron vs. Delta and −12.8 (95% CI −13.2 to −12.5) for later Omicron vs. Delta. Collectively, more data using the same inclusion criteria from a wider range of different healthcare systems with age-stratified comparisons of in-hospital mortality during different variant waves was deemed to be warranted.

**Added value of this study**

We conducted a cohort study of age-stratified 28-day in-hospital mortality in adult patients hospitalised with SARS-CoV-2 Wild-type, Alpha, Delta, or Omicron infection across 10 centres in 9 countries: Germany, Italy, Kenya, Lithuania, Mexico, Poland, Portugal, Sweden, and United Kingdom. We found age to be an important effect measure modifier of the risk of 28-day in-hospital mortality and thus presented results by age groups (18–49 years, 50–69 years, ≥70 years). The cumulative incidence of mortality decreased throughout the study period, particularly during the Omicron period. However, this trend was not as apparent in regression models adjusted for age, sex, calendar time, and comorbidities. Furthermore, results differed in analyses by COVID-19 vaccination status and different countries. When comparing Omicron sublineages, BA.1 carried a higher risk of mortality than that seen with the more recently circulating Omicron sublineages BA.2 and BA.5. Given the scarcity of studies investigating trends of age-stratified in-hospital mortality from multiple countries and settings, this study of 38,585 participants (19,763 Wild-type, 6387 Alpha, 3640 Delta, and 8795 Omicron) provides an important contribution to the existing literature.

**Implications of all the available evidence**

Collectively, in-hospital mortality rates have decreased over the course of the pandemic, particularly since the emergence of the Omicron variant. The results from our multinational cohort study suggest that the observed decrease in in-hospital mortality seems to reflect a combined effect of immunity from vaccinations and previous infections. Also, it cannot be excluded that differences in virulence between SARS-CoV-2 variants may have contributed. However, the detrimental effects of Omicron observed in the Hong Kong population with low population immunity and poor vaccine uptake among elderly and frail individuals suggest a high virulence of Omicron in this population. Taken together, the available evidence reinforces the importance of sustained efforts to protect elderly and frail individuals from severe outcomes with adequate protective measures.

## Introduction

Since the first cases of COVID-19 were reported in Wuhan, China at the end of 2019, more than 750 million confirmed cases and nearly 7 million deaths have been reported to the World Health Organization (WHO).^1^ The SARS-CoV-2 virus has exhibited major changes in its phenotypic properties over time, including altered transmissibility, antigenicity, and immune escape.^2^ Consequentially, the WHO has so far declared five variants of concern (VOC): Alpha, Beta, Gamma, Delta, and Omicron.^3^ The Omicron variant, the latest VOC to be declared, was detected in South Africa in November 2021, and has, unlike preceding variants, evolved into numerous sublineages such as BA.1, BA.2, BA.4, and BA.5 as well as a continuously evolving plethora of BA.2 and BA.5 descendants and recombinants.^2^^,^^4^ Furthermore, compared to preceding variants, the Omicron variant has been claimed to have higher transmissibility but less severe clinical progression and lower risk of mortality.^5^^,^^6^ Despite this, COVID-19 associated hospitalisation rates have been reported to be high also during the initial surge of the Omicron variant, although the proportion of patients who are treated primarily for COVID-19 have decreased.^7^^,^^8^ Previous studies from Hong Kong have demonstrated the detrimental effects Omicron can cause in populations with low population immunity and poor vaccine uptake among elderly and frail individuals.^9^^,^^10^ Furthermore, given the sheer number of individuals who have been infected with Omicron, even a lower mortality rate has resulted in a substantial absolute number of deaths.

Understanding characteristics and outcomes of patients hospitalised with COVID-19 throughout the pandemic is crucial to guide clinical management and to understand and plan future COVID-19 associated resource allocation. COVID-19 in-hospital mortality rates in the US have ranged from above 20% during the early phases to below 10% since the emergence of the Omicron variant.^11^ Despite these lower mortality rates, the Omicron variant was in a Swiss study associated with a significantly increased risk of in-hospital mortality when compared with influenza.^12^ Results from the US have demonstrated in-hospital mortality to be 0.69 (95% confidence interval [CI] 0.68–0.70) as likely during the early Omicron period and 0.24 (95% CI 0.22–0.25) during the later Omicron period compared with the Delta period.^13^ However, more data from a wider range of different healthcare systems and hospital settings are warranted to better understand morbidity and mortality burden in patients hospitalised with COVID-19 during different variant waves. Furthermore, age has repeatedly been demonstrated to have a decisive impact on COVID-19 mortality rates, but this has varied substantially across different geographical areas and during different periods.

The European Cohorts of Patients and Schools to Advance Response to Epidemics (EuCARE) project is a multinational collaborative effort aiming to support effective responses to the COVID-19 epidemic with a strong emphasis on emerging SARS-CoV-2 variants (website www.eucareresearch.eu). Within the project, the EuCARE-HOSPITALISED study (ClinicalTrials.gov ID NCT05463380) aims to analyse the clinical course of patients hospitalised with SARS-CoV-2 infection caused by different variants.^14^ In this study, we aimed to compare 28-day in-hospital mortality in adult patients hospitalised with COVID-19 during the Wild-type, Alpha, Delta, and Omicron variant waves over the period February 2020–October 2022, and to investigate whether age was an effect measure modifier. Furthermore, the risk of in-hospital mortality associated with BA.1, BA.2, and BA.5 Omicron sublineages was also compared.

## Methods

### Study design and data sources

We conducted a cohort study of 28-day in-hospital mortality for different SARS-CoV-2 variants in adult patients hospitalised with COVID-19 across 10 centres in 9 countries: Germany, Italy, Kenya, Lithuania, Mexico, Poland, Portugal, Sweden, and United Kingdom. Data collection was performed both retrospectively and prospectively and uploaded to the EuCARE database within the EuCARE-HOSPITALISED study. The raw data were derived from electronic health record systems, health registries, and other information systems, as described in Supplementary Text S1.

### Study population

The study population included adult patients (≥18 years) who were hospitalised with COVID-19 or died in the emergency department within one day of arrival without being hospitalised. Being hospitalised with COVID-19 was defined as testing positive for SARS-CoV-2 by polymerase chain reaction (PCR) or antigen (only for the Kenyan centre) within 14 days prior to admission or during the hospitalisation. Hospitalisations with an admission any time from 1 February 2020 and a discharge up until 15 October 2022 were considered. In case of multiple admissions, the first hospitalisation or ED visit per participant which met these criteria was included.

### SARS-CoV-2 variants

The following SARS-CoV-2 variants were considered as our main exposure of interest: Wild-type, Alpha, Delta, and Omicron. The two other declared VOCs, Beta and Gamma, were not considered due to the low number of patients. SARS-CoV-2 variant classification was based on results from sequencing of the whole genome, multiple genomic regions, or the spike gene, or if no sequencing had been performed, inferred from date of hospital admission and country of the site of enrolment. For participants without sequence information, the variant was inferred from the 7-day moving average national distribution of variants, derived from metadata from whole genome sequences retrieved from Global Initiative on Sharing All Influenza Data (GISAID). Two different cut-offs were used to infer variants: >75% (i.e., >75% of GISAID sequences in the specific 7-days window and country belonged to the assigned variant) and >90%. The >75% cut-off was used in the main analyses and the >90% in a sensitivity analysis as described in the Statistical Methods. If none of the considered variants were exceeding the specified cut-offs, the SARS-CoV-2 variant was scored as unclassified, and the patient was excluded from all analyses.

### Omicron sublineages

To investigate whether in-hospital mortality differed across Omicron sublineages, we compared the risk among participants hospitalised with a BA.1, BA.2, and BA.5 infection. BA.3 and BA.4 sublineages, as well as BA.2 and BA.5 descendants, were not included in these analyses due to the very low number of identified participants (less than 50 for both sublineages).

### Study outcome and other collected variables

The study outcome was the time to all-cause 28-day in-hospital mortality, defined as the time from the date of hospital admission up until the date of death or hospital discharge or 28 days of hospitalisation (at which time follow-up was censored), whichever occurred first. No participants were lost to-follow-up and the follow-up of participants who were still hospitalized after 28 days was right-censored. For all centres, discharge meant discharge from all units within the included hospitals. As such the patient could be discharged to their home, to another hospital or to another care facility not covered by the data sources. This is further described in Supplementary Text S1. Data on reason for hospital discharge was not available in the dataset.

Data were also collected for age, biological sex, comorbidities, COVID-19 vaccinations, and vital signs and laboratory values at admission, using a uniform electronic case report form. Data on the following comorbidities, which are suggested to be associated with an increased risk of severe COVID-19, were collected: cancer, cardiac or cerebrovascular disease, chronic kidney disease, chronic liver disease, chronic lung disease, diabetes (type 1 or 2), hypertension, immunocompromised state, neurologic conditions, and obesity.^15^ COVID-19 vaccinations were considered up until 14 days before the date of hospital admission. Vital signs and laboratory values included respiratory rate, peripheral oxygen saturation, C-reactive protein, white blood cell count, platelet count, and lymphocyte count.

### Statistical methods

First, we described baseline characteristics of the Wild-type, Alpha, Delta, and Omicron variant groups. Continuous variables were presented as median (interquartile range [IQR]) and categorical variables were reported as frequencies (percentage). Characteristics of the variant exposure groups were compared with the Kruskal–Wallis tests for continuous variables and the Chi squared tests for categorical variables. Baseline characteristics were also presented for unvaccinated participants, vaccinated (≥2 doses) participants in the Delta and Omicron groups, and participants carrying the Omicron BA.1, BA.2, and BA.5 sublineages.

For analyses of in-hospital mortality, we used a competing risks-based approach, including cumulative incidence functions (CIF) and Fine–Gray subdistribution hazard models. The CIF describes the incidence of the occurrence of 28-day in-hospital mortality while taking the competing event, discharged alive, into account.^16^ This was done in the framework of a Fine–Gray subdistribution hazard model which is typically considered a CIF regression model.^17^ Since we did not have data on reason for hospital discharge, e.g., complete recovery from the infection, transfers to other healthcare facilities due to hospital capacity constraints, or deterioration warranting care in another hospital, we considered hospital discharge to lead to informative censoring and a competing event (e.g., in the case of participants transferred to a nursing home or to another hospital) and consequently decided to use the Fine–Gray subdistribution hazard model framework.

At the outset of the study, we hypothesized that age was an effect measure modifier for the comparisons between the Wild-type, Alpha, Delta, and Omicron exposure groups. We therefore formally tested for interaction in the adjusted Fine–Gray subdistribution model (described in detail below) by including an interaction parameter for age as a categorical variable (18–49 years, 50–69 years, and ≥70 years) and variant as a categorical variable. The age groups were choses on the basis of clinical judgement and increases in COVID-19 infection-fatality ratio with age as previously reported.^18^ There was strong evidence to support interaction between age and variant from using the log-likelihood ratio rest (type 3 interaction *P* = 0.0005), and results for the main analysis throughout the manuscript were therefore presented using these age groups.

Below, we provide more details about how the Fine–Gray subdistribution models were fitted in each of the age groups. First, because there was evidence that the proportional hazards assumption was violated for the variable centre, we used stratification (i.e., a Fine–Gray subdistribution hazards model stratified by centre). Besides effect measure modification by age, we also tried to minimise potential confounding bias. The adjusted models included age (as a continuous variable), sex, calendar time, and all studied comorbidities (cancer, cardiac or cerebrovascular disease, chronic kidney disease, chronic liver disease, chronic lung disease, diabetes (type 1 or 2), hypertension, immunocompromised state, neurologic conditions, and obesity) which were all fitted as binary covariate. Calendar time was included in the models to try to separate the inherent effects of the virus variants from other changes occurring during the pandemic. This was modelled with restricted cubic splines, with knots set at the time when data on the use of Dexamethasone emerged from the RECOVERY trial (1 July 2020), when receipt of two doses started to be readily available (1 July for ≥70 years, 1 September 2021 for 50–69 years, and 1 October 2021 for 18–49 years), and after the large Omicron surge in Europe, when a large majority of the population had been infected at least once (9 March 2022).^19^ To show how the inclusion of calendar time in the models affected the estimates, we present models for the main analysis with and without inclusion of calendar time.

We also tried to minimise the confounding effect of vaccination by performing subgroup analyses by vaccination status. The first included participants with no history of vaccination up to 14 days before the hospital admission, whereas the second included participants who had received at least two doses of COVID-19 vaccine. Both vaccination analyses were restricted to participants with complete data on vaccination and the latter was done only for the Delta and Omicron comparison since receipt of two doses or more was not available during the entire Wild-type and Alpha periods.

For the main analysis, a total of 6 sensitivity analyses were performed, of which 5 were based on the primary >75% cut-off for variant classification: i) after including only centres who contributed more than 50 participants in each variant group; ii) after restricting to participants without a sequenced SARS-CoV-2 variant; iii) after restricting to participants with a positive SARS-CoV-2 test any time from 14 days before to 1 day after the hospital admission; iv) after including only participants from Sweden, v) after including only participants from outside of Sweden, and vi) after restricting to participants classified as Wild-type, Alpha, Delta, or Omicron when using a cut-off of >90% instead of >75%. In this last sensitivity analysis, participants who could not be classified as Wild-type, Alpha, Delta, or Omicron based on this cut-off were excluded.

COVID-19 vaccination status was missing for 2% (n = 709) of the study population. These participants were excluded from analyses taking vaccination status into account. None of the other independent variables included in the regression models (age, sex, comorbidities, and day of hospital admission) had missing values. Vital signs and laboratory values showed a larger proportion of missing data (ranging from 8% to 60%). We used this data for descriptive analyses only, and used a complete case approach to handle missing data (i.e., median [IQR] values were only presented for participants with data available), and the number and percentage of participants with missing values described for each variant exposure group.

For the secondary analysis, comparing the hazard of the 28-day in-hospital mortality according to Omicron sublineages, the study was not powered to detect an interaction with age and therefore overall results are presented. These models also included COVID-19 vaccination status, since vaccination with three or more doses were readily available during this time period.

Pairwise comparisons were made for Wild-type vs. Alpha, Wild-type vs. Delta, Alpha vs. Delta, Wild-type vs. Omicron, Alpha vs. Omicron, Delta vs. Omicron (main analysis) and BA.1 vs. BA.2, BA.1 vs. BA.5, and BA.2 vs. BA.5 (secondary analysis). All pairwise comparisons were obtained from a single model (the software automatically changed the reference group to provide the estimates for the different contrasts of interest).

All data processing and descriptive analyses were conducted using R version 4.1.0. All cumulative incidence analyses and regression modelling were conducted using SAS version 9.4 (Carey NC USA).

### Role of the funding source

The funder of the study had no role in the study design, data collection, data analysis, data interpretation, writing of the report, or decision to submit the paper for publication.

## Results

### Study population

A total of 39,297 SARS CoV-2-infected hospitalised participants were considered for analysis (Figure S1). For 2% (n = 712) of these, the acquired variant could not be classified as one of the variants of interest (Wild-type, Alpha, Delta, or Omicron) and these participants were therefore excluded from the study analyses. The final study population consisted of 38,585 participants with the following variant distribution: 19,763 Wild-type, 6387 Alpha, 3640 Delta, and 8795 Omicron. Overall, for 14% (n = 5582) of the study population the variant was assigned based on sequence data while for the remaining 33,003 participants the variant was estimated based on the national variant distribution from GISAID (see Methods) at the date of hospital admission (Figure S2). The proportion included based on a sequenced sample was 3% (n = 677) for the Wild-type group, 17% (n = 1099) for the Alpha group, 30% (n = 1103) for the Delta group, and 31% (n = 2703) for the Omicron group. When using a cut-off of >90% instead of >75% for SARS-CoV-2 variants, 37,536 participants were included: 21,296 Wild-type, 4354 Alpha, 3441 Delta, and 8445 Omicron. A total of 1,572, 2,399, and 2303 participants were considered infected with the BA.1, BA.2, and BA.5 Omicron variant, respectively. Data on national distributions of Omicron sublineages from the GISAID metadata are shown in Figure S3.

### Baseline characteristics

The median (IQR) age at hospital admission was 65 (52–78) years in the Wild-type, 62 (50–73) in the Alpha, 60 (44–75) in the Delta, and 72 (52–82) in the Omicron group (Table 1). The percentage of males ranged from 50% (n = 4417) in the Omicron group to 60% (n = 11,756) in the Wild-type group. Hypertension was the most common comorbidity in all variant groups, ranging from 34% (n = 1236) in the Delta group to 51% (n = 4474) in the Omicron group. A total of 18% (n = 1553) of participants were immunocompromised in the Omicron group compared with 7% (n = 1432) in the Wild-type group, 7% (n = 425) in the Alpha group, and 8% (n = 283) in the Delta group. Among participants with COVID-19 vaccination data available (n = 37,876), 100% (n = 19,524) in the Wild-type were unvaccinated before the hospitalisation, compared with 93% (n = 5718) in the Alpha, 65% (n = 2303) in the Delta, and 23% (n = 2023) in the Omicron groups. Among those with a C-reactive protein measurement available (n = 34,475), the Omicron group had the lowest values (median 29 mg/L, IQR 7–89), whereas the Alpha group had the highest values (median 66 mg/L, IQR 23–127).

**Table 1:** Characteristics of the SARS-CoV-2 variant groups.

Variable	Overall (n = 38,585)	Wild-type (n = 19,763)	Alpha (n = 6387)	Delta (n = 3640)	Omicron (n = 8795)
**Age, years**	65.0 [51.0, 78.0]	65.0 [52.0, 78.0]	62.0 [50.0, 73.0]	60.0 [44.0, 75.0]	72.0 [52.0, 82.0]
18–49	9099 (23.6)	4297 (21.7)	1596 (25.0)	1198 (32.9)	2008 (22.8)
50–69	13,286 (34.4)	7251 (36.7)	2751 (43.1)	1218 (33.5)	2066 (23.5)
70 years or older	16,200 (42.0)	8215 (41.6)	2040 (31.9)	1224 (33.6)	4721 (53.7)
**Male sex**	21,831 (56.6)	11,756 (59.5)	3682 (57.6)	1976 (54.3)	4417 (50.2)
**Comorbidities**					
Cancer	3129 (8.1)	1363 (6.9)	334 (5.2)	250 (6.9)	1182 (13.4)
Cardiac or cerebrovascular disease	10,198 (26.4)	5084 (25.7)	1162 (18.2)	785 (21.6)	3167 (36.0)
Chronic kidney disease	4512 (11.7)	2066 (10.5)	480 (7.5)	425 (11.7)	1541 (17.5)
Chronic liver disease	981 (2.5)	467 (2.4)	120 (1.9)	95 (2.6)	299 (3.4)
Chronic lung disease	5645 (14.6)	2776 (14.0)	775 (12.1)	466 (12.8)	1628 (18.5)
Diabetes	7863 (20.4)	4271 (21.6)	1029 (16.1)	654 (18.0)	1909 (21.7)
Hypertension	15,846 (41.1)	7958 (40.3)	2178 (34.1)	1236 (34.0)	4474 (50.9)
Immunocompromised	3693 (9.6)	1432 (7.2)	425 (6.7)	283 (7.8)	1553 (17.7)
Neurologic conditions	3233 (8.4)	1543 (7.8)	265 (4.1)	274 (7.5)	1151 (13.1)
Obesity	6829 (17.7)	3627 (18.4)	1302 (20.4)	532 (14.6)	1368 (15.6)
Number of comorbidities	1.0 [0.0, 3.0]	1.0 [0.0, 2.0]	1.0 [0.0, 2.0]	1.0 [0.0, 2.0]	2.0 [1.0, 3.0]
0	11,932 (30.9)	6252 (31.6)	2498 (39.1)	1374 (37.7)	1808 (20.6)
1	9047 (23.4)	4768 (24.1)	1632 (25.6)	853 (23.4)	1794 (20.4)
2	7345 (19.0)	3812 (19.3)	1067 (16.7)	629 (17.3)	1837 (20.9)
3	5314 (13.8)	2634 (13.3)	683 (10.7)	432 (11.9)	1565 (17.8)
4 or more	4947 (12.8)	2297 (11.6)	507 (7.9)	352 (9.7)	1791 (20.4)
**COVID-19 vaccine doses**^a^					
Unvaccinated	29,568 (78.1)	19,524 (99.9)	5718 (93.0)	2303 (64.8)	2023 (23.5)
1 dose	818 (2.2)	25 (0.1)	322 (5.2)	266 (7.5)	205 (2.4)
2 doses	2814 (7.4)	3 (0.0)	109 (1.8)	903 (25.4)	1799 (20.9)
3 doses or more	4676 (12.3)	0 (0.0)	1 (0.0)	82 (2.3)	4593 (53.3)
**Admission vitals and lab values**					
Respiratory rate, breaths/minute	24.0 [20.0, 29.0]	24.0 [20.0, 30.0]	24.0 [21.0, 30.0]	23.0 [18.0, 29.0]	22.0 [20.0, 27.0]
Missing data	12,564 (32.6)	6657 (33.7)	2275 (35.6)	1454 (39.9)	2178 (24.8)
Peripheral oxygen saturation, %	93.0 [89.0, 95.0]	92.0 [88.0, 95.0]	92.0 [89.0, 95.0]	93.0 [90.0, 96.0]	94.0 [91.0, 96.0]
Missing data	11,720 (30.4)	5800 (29.3)	2266 (35.5)	1448 (39.8)	2206 (25.1)
C-reactive protein, mg/L	54.0 [13.8, 118.0]	63.0 [20.0, 126.8]	66.0 [23.0, 127.3]	39.0 [8.2, 101.1]	29.0 [6.7, 89.0]
Missing data	4110 (10.7)	1827 (9.2)	568 (8.9)	400 (11.0)	1315 (15.0)
White blood cell count, 10^9^ cells/L	7.2 [5.2, 9.8]	7.0 [5.2, 9.6]	6.4 [4.8, 8.7]	6.9 [4.9, 9.3]	8.4 [6.2, 11.2]
Missing data	3636 (9.4)	1780 (9.0)	523 (8.2)	346 (9.5)	987 (11.2)
Platelet count, 10^9^ cells/L	202.0 [156.0, 262.0]	204.0 [158.0, 263.0]	195.0 [153.0, 253.0]	194.0 [148.0, 251.0]	207.0 [159.0, 271.0]
Missing data	4746 (12.3)	2231 (11.3)	750 (11.7)	695 (19.1)	1070 (12.2)
Lymphocyte count, 10^9^ cells/L	1.0 [0.7, 1.4]	1.0 [0.7, 1.4]	1.0 [0.7, 1.4]	1.0 [0.7, 1.4]	1.1 [0.7, 1.6]
Missing data	12,475 (32.3)	4996 (25.3)	1326 (20.8)	855 (23.5)	5298 (60.2)

Note: When comparing the four groups, *P* values from Kruskal–Wallis tests and Chi squared tests were <0.001 for all variables.

Abbreviation: COVID-19 = Coronavirus disease 2019.

a 709 participants (2%) were excluded from the analyses due to unknown vaccination status before the hospitalisation.

Characteristics of participants unvaccinated before the hospitalisation are presented in Table S1. A total of 41.4% (n = 837) of unvaccinated Omicron participants were ≥70 years and 67% (n = 1360) had any of the studied comorbidities. Characteristics for participants in the Delta and Omicron groups having received two doses or more before the hospitalisation were similar (Table S2).

Characteristics of the participants carrying the Omicron sublineages are presented in Table S3. The median (IQR) age was 67 (44–80) years in the BA.1, 73 (57–83) years in the BA.2, and 76 (62–84) years in the BA.5 sublineage. Among participants with COVID-19 vaccination data available (n = 6138), 41% (n = 630) in the BA.1 were unvaccinated before the hospitalisation, compared to 15% (n = 347) in the BA.2, and 17% (n = 382) in the BA.5 Omicron group.

### 28-Day in-hospital mortality in the wild-type, Alpha, Delta, and Omicron exposure groups

The overall 28-day in-hospital mortality rates were 14% (n = 2746) in the Wild-type, 10% (n = 628) in the Alpha, 9% (n = 312) in the Delta, and 6% (n = 534) in the Omicron groups. A total of 8% (n = 1611) in the Wild-type group had not died or been discharged alive by 28 days and were thus censored. This proportion was 6% (n = 358) in the Alpha group, 8% (n = 280) in the Delta group, and 5% (n = 421) in the Omicron group. A formal test for interaction between age and variant in the adjusted Fine–Gray subdistribution hazard model carried a very small *P* value (*P* < 0.0001), providing strong evidence that age was an effect measure modifier for the association of interest.

**Fig. 1:**
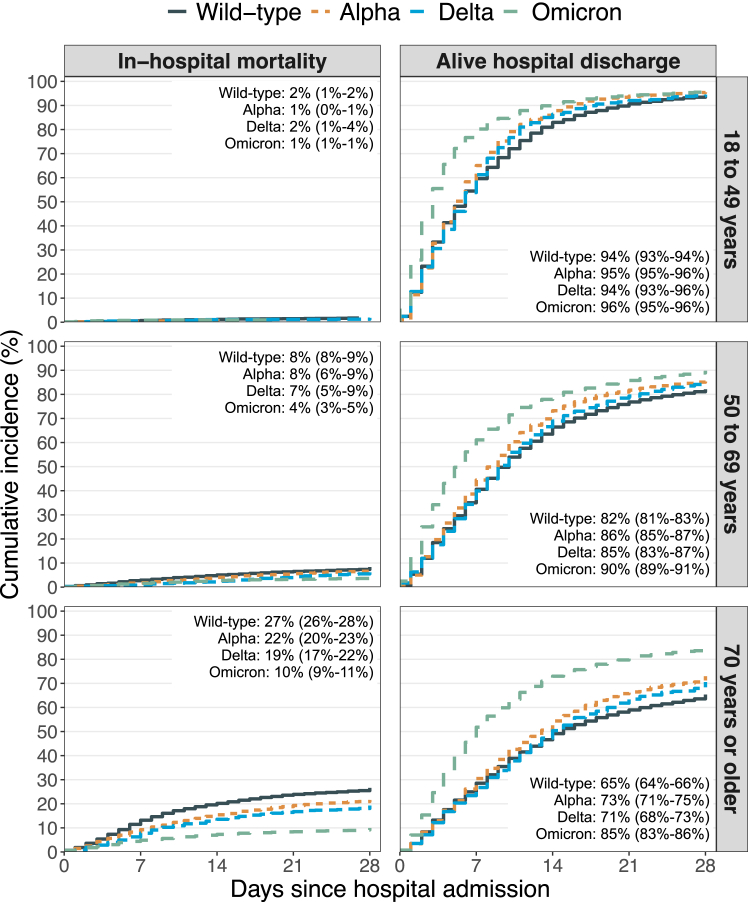
**Age-stratified 28-day cumulative incidence of in-hospital mortality and alive hospital discharge**. **Note:** The text in the figure describes the cumulative incidence at 28 days except for in-hospital mortality among participants 18–49 years in the Wild-type group (day 27) Alpha group (day 16), and Omicron group (day 27).

Age-stratified 28-day cumulative incidences of in-hospital mortality and alive hospital discharge in the Wild-type, Alpha, Delta, and Omicron groups are presented in Fig. 1. The cumulative incidences of mortality ranged from 1% to 2% among participants 18–49 years, 4%–8% among participants 50–69 years, and 10%–27% among participants 70 years or older. The cumulative incidences of mortality among participants aged 70 years or older was 27% (26%–28%) in the Wild-type group, 22% (20%–23%) in the Alpha group, 19% (17%–22%) in the Delta group, and 10% (9%–11%) in the Omicron group. The Omicron group were discharged alive from the hospital faster than the other exposure groups across all age three groups.

**Fig. 2:**
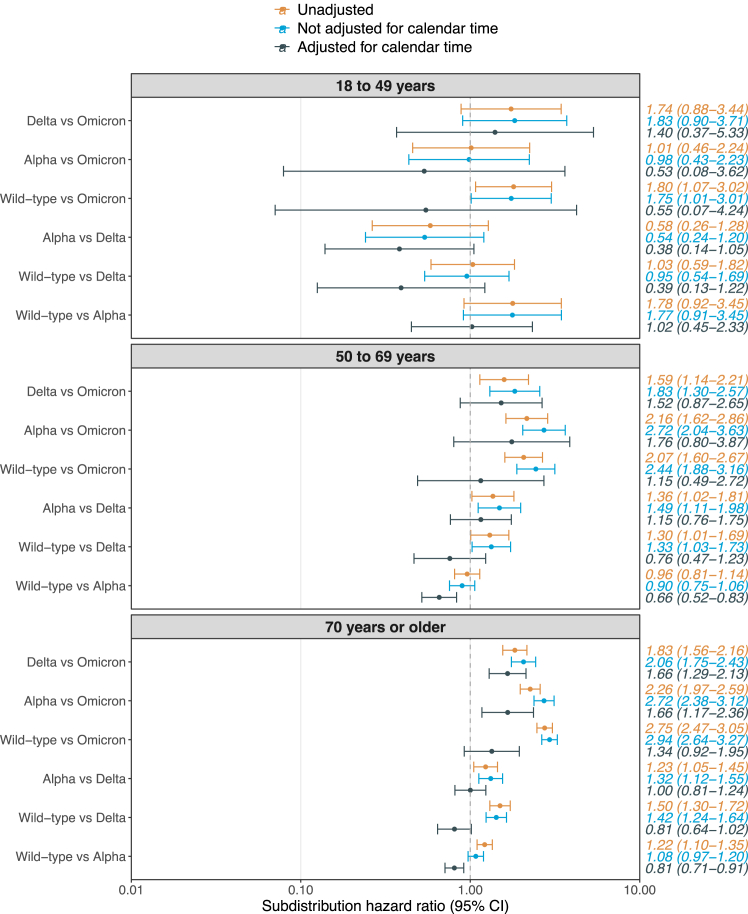
**Subdistribution hazard ratios for pairwise comparisons of 28-day in-hospital mortality**. **Note:** Both adjusted models were adjusted for age, sex, and all studied comorbidities. Centre was included as a stratification factor in all models. **Abbreviation:** CI=Confidence interval.

Fig. 2 presents unadjusted and adjusted subdistribution hazard ratios (SHRs) (95% CI) stratified by age for the pairwise comparisons of age-stratified 28-day in-hospital mortality according to variants. Large differences between the unadjusted and fully adjusted SHRs were observed, with all fully adjusted point estimates being lower than the unadjusted point estimates. These differences seemed to be mainly attributed to the adjustment for calendar time, with large differences in adjusted analysis with vs. without inclusion of calendar time. The statistical precision increased with increasing age and no sufficient evidence against the null hypothesis of no difference in risk when comparing the variants could be observed for participants aged 18–49 years. A reduced risk of in-hospital mortality was observed for the Wild-type variant vs. the Alpha variant among participants 50–69 years (adjusted SHR 0.66 [0.52–0.83]). This was also observed among participants ≥70 years (adjusted SHR 0.81 [0.71–0.91]). Furthermore, an increased risk of in-hospital mortality was observed for Alpha vs. Omicron (adjusted SHR 1.66 [1.17–2.36]) and Delta vs. Omicron (adjusted SHR 1.66 [1.29–2.13]) among participants ≥70 years. Similar trends, albeit not statistically significant, were also observed among participants 50–69 years.

**Fig. 3:**
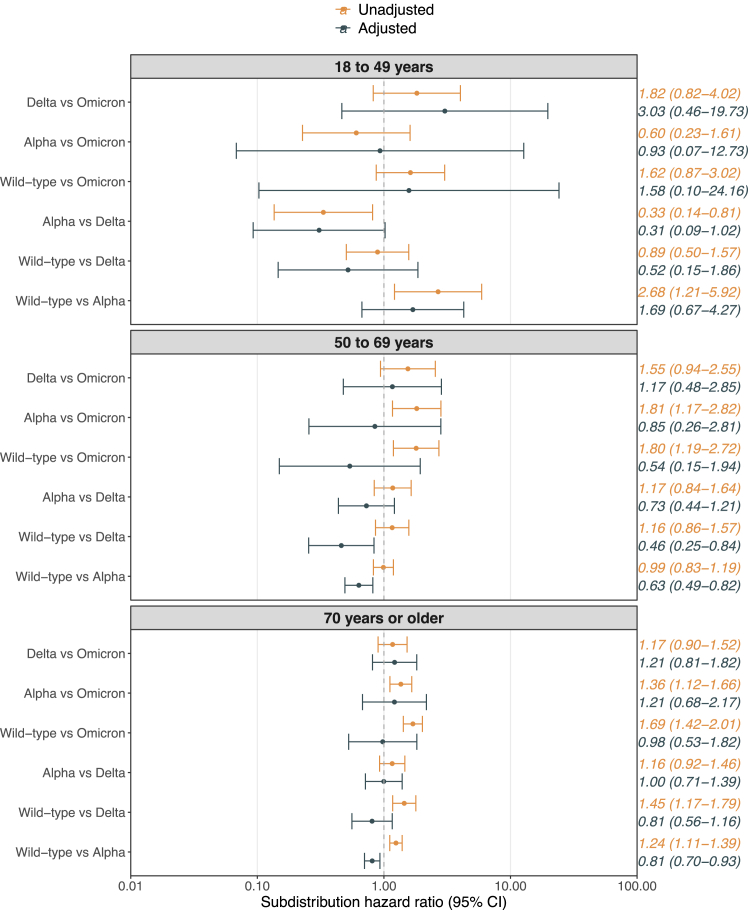
**Subdistribution hazard ratios for pairwise comparisons of 28-day in-hospital mortality among unvaccinated participants**. **Note:** The adjusted model was adjusted for age, sex, calendar time, and all studied comorbidities. Centre was included as a stratification factor in all models. **Abbreviation:** CI=Confidence interval.

Unadjusted and adjusted SHRs (95% CI) for unvaccinated participants are presented in Fig. 3. A reduced risk of in-hospital mortality was also observed for the Wild-type variant vs. the Alpha variant among participants 50–69 years and ≥70 years. A statistically significant increased risk in the Alpha and Delta groups vs. the Omicron group could not be observed. The cumulative incidences (95% CI) of in-hospital mortality among unvaccinated patients ≥70 years were 27% (26%–28%) in the Wild-type cohort, 22% (20%–24%) in the Alpha cohort, 19% (16%–23%) in the Delta cohort, and 17% (15%–21%) in the Omicron cohort (Figure S4).

For the vaccinated Delta and Omicron groups, an increased risk of in-hospital mortality was observed for the Delta vs. Omicron group among participants >70 years (adjusted SHR 2.11 [1.49–3.00]) (Figure S5). No such significant difference could be observed among participants 50–69 years (adjusted SHR 1.81 [0.75–4.39]), and no participant 18–49 years died in the Delta group.

Results for the six sensitivity analyses for the main analysis, described in the Methods section, are presented in Figures S6–S11 in the Supplement. The unadjusted and adjusted SHRs were similar for the analysis including only centres who contributed more than 50 participants in each variant group (Figure S6), and when restricting the analysis to participants without a sequenced SARS-CoV-2 variant (Figure S7). A total of 28,455 participants were included in the analysis restricted to participants with a positive SARS-CoV-2 test any time from 14 days before to 1 day after the hospital admission: 14,660 Wild-type, 4556 Alpha, 2383 Delta, and 6856 Omicron. Results were generally similar also for this analysis compared with the main analysis (Figure S8). SHRs after including only participants from Sweden vs. including only participants from outside of Sweden are presented in Figure S9. The SHRs were rather similar for participants ≥70 years, whereas the SHRs for participants 50–69 years differed to a higher extent. A significantly reduced risk of in-hospital mortality could be observed for Wild-type vs. Alpha (adjusted SHR 0.34 [0.21–0.55]) and Wild-type vs. Delta (adjusted SHR 0.20 [0.09–0.43]) in Sweden, which was not observed for the other countries (adjusted SHRs 0.80 [0.60–1.07] and 1.14 [0.59–2.19]). In-hospital mortality rates by centre, variant exposure groups, and age groups are presented in Figure S10. Large differences in mortality rates could be observed across the different centres. Finally, the unadjusted and adjusted SHRs were similar for the analysis using a cut-off of >90% instead of >75% for the variant exposure groups (Figure S11).

### 28-Day in-hospital mortality in the Omicron BA.1, BA.2, and BA.5 groups

**Fig. 4:**
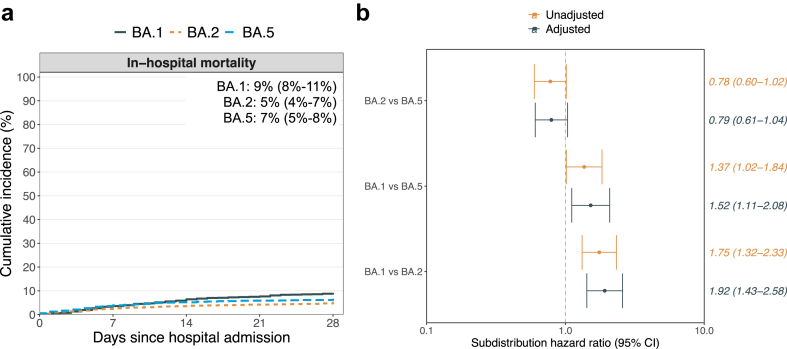
**Cumulative incidence and subdistribution hazard ratios for 28-day in-hospital mortality in the Omicron sublineages groups**. **Note:** Panel A shows the cumulative incidence and panel B shows the subdistribution hazard ratios. The text in the figure describes the cumulative incidence (95% confidence interval) at 28 days. The adjusted model was adjusted for age, sex, all studied comorbidities, and COVID-19 vaccination status before the hospitalisation. Centre was included as a stratification factor in all models. **Abbreviation:** CI=Confidence interval.

The 28-day cumulative incidence of in-hospital mortality was 9% (8%–11%) in the BA.1, 5% (4%–7%) in the BA.2, and 7% (5%–8%) in the BA.5 sublineage (Fig. 4a). Among the 6274 participants included in these analyses, 397 participants (6%) died within 28 days, of which 16 (4%) were 18–49 years, 57 (14%) were 50–69 years, and 324 (82%) were ≥70 years. Fig. 4b presents unadjusted and adjusted subdistribution hazard ratios (SHRs) (95% CI) for the pairwise comparisons of in-hospital mortality according to sublineages. Unadjusted and adjusted SHR for in-hospital mortality were similar for all comparisons. An increased risk of in-hospital mortality was observed for the BA.1 vs. BA.2 group (adjusted SHR 1.92 [1.43–2.58]) as well as for the BA.1 vs. BA.5 group (adjusted SHR 1.52 [1.11–2.08]). The adjusted SHR (95% CI) was 0.79 (0.61–1.04) for the BA.2 vs. BA.5 group.

## Discussion

In this multinational cohort study of hospitalised COVID-19 patients in nine countries within the EuCARE project, we found age to be an important effect measure modifier of the risk of 28-day in-hospital mortality. The unadjusted cumulative incidence of mortality decreased throughout the study period, particularly during the Omicron period. However, this trend was not as apparent in regression models adjusted for age, sex, calendar time, and comorbidities. Furthermore, results differed in analyses by COVID-19 vaccination status and different countries. When comparing Omicron sublineages, BA.1 carried a higher risk of mortality than that seen with the more recently circulating Omicron sublineages BA.2 and BA.5.

We found the risk of in-hospital mortality to increase substantially with age, a finding which is in line with an extensive body of evidence.^13^^,^^18^^,^^20^^,^^21^ This reinforces the importance of sustained efforts to protect the elderly and frail individuals with adequate protective measures. The reduced risks of in-hospital mortality for Omicron variant infected participants compared with Alpha and Delta observed in our study are in line with previous studies. A study from the US, comparing the in-hospital mortality during the Delta (July to October 2021), early Omicron (January to March 2022), and later Omicron (April to June 2022) periods found the adjusted mortality risk difference to be −5.3 (95% CI −5.5 to −5.0) for early Omicron vs. Delta and −12.8 (95% CI −13.2 to −12.5) for later Omicron vs. Delta.^13^ These results are consistent with our findings, showing a higher hazard of in-hospital mortality for participants infected with Delta vs. Omicron as well as for both BA.2 and BA.5 compared with BA.1. We extended these analyses by performing subgroup analyses of unvaccinated and vaccinated (two or more doses) patients, observing no statistically significant difference between Delta and Omicron for unvaccinated participants, but an increased risk of in-hospital mortality for Delta vs. Omicron among vaccinated participants. Previous studies from Hong Kong have shown the detrimental effects Omicron can cause in populations with low population immunity and poor vaccine uptake among elderly and frail individuals.^9^^,^^10^

As mentioned previously, our findings of increased risk of mortality in hospitalised patients infected with BA.1 compared with BA.2 and BA.5 are in line with previous studies.^13^ A large cohort study from England, including 984,337 individuals infected with BA.1 and 258,875 infected with BA.2 found BA.2 to have a lower risk of death compared with BA.1 (HR = 0.80, 95% CI 0.71–0.90).^22^ Furthermore, a South African study found patients infected with BA.1 to have a higher odds of mortality compared with the BA.4/BA.5 group (adjusted odds ratio 1.3, 95% CI 1.2–1.4).^23^

Strengths of our study include the same inclusion criteria for patients hospitalised with COVID-19 across multiple countries, primarily in Europe, but also outside of Europe. This favours external validity by investigating the overall effects of SARS-CoV-2 variants across a wide range of countries and continents. The internal validity is also strengthened by using the same inclusion criteria and a common case report form across all participating centres. Furthermore, we collected data on a wide range of underlying health conditions known to increase the risk of severe COVID-19, as well as data on number of COVID-19 vaccine doses received before the hospitalisation.^15^

Limitations of the study include the different possibilities and capacities of centres to include data from the entire study period, possibly affecting the observed estimates for the different variants. However, we performed a sensitivity analysis after including only the centres providing >50 participants for each of the variants and the results were similar. Furthermore, a total of 58% of the study population was from the centre KI, meaning that the data from Stockholm, Sweden had the strongest impact on the results, thus possibly reducing the generalisability of our findings to other healthcare systems. Another limitation includes the fact that viral variant was inferred for most of the participants, and the number of participants with a sequenced variant available were too few to perform analyses restricted to these participants. However, results were similar after excluding this latter group. Similarly, due to a limited sample size for each age group across the Omicron sublineages, these analyses were not shown age-stratified, but rather age was adjusted for in the regression models. Data on reason of hospital discharge was not available. As such, we could not ensure that hospital discharge represented complete recovery from the infection, transfers to other healthcare facilities due to hospital capacity constraints, or deterioration warranting care in another hospital. On a similar note, the study outcome was 28-day all-cause in-hospital mortality. Patients could therefore have died from causes not directly related to COVID-19, and as such the observed differences in in-hospital mortality might have not been solely influenced by the COVID-19 disease severity but also other disease processes. Our inclusion criteria were broad, including all patients with a positive SARS-CoV-2 test from 14 days before hospital admission to the day of hospital discharge. This means that differences in testing procedures such as SARS-CoV-2 screening tests and hospital admission procedures across different countries could affect the case mix of included patients. Differences in case-mix over time should however be partly mitigated by controlling for calendar time in the models. It is further difficult to ascertain whether the patients were hospitalised with or due to COVID-19 and data on respiratory function was too limited to perform a sensitivity analysis restricted to those who were hospitalised with pneumonia. However, these broader inclusion criteria were used with the aim of focusing on a larger range of clinical presentations of SARS-CoV-2 infected hospitalised patients. Furthermore, results were similar after restricting the analysis to participants who had tested positive for SARS-CoV-2 in close relation to the hospital admission (excluding nosocomial infections). Finally, many centres were not able to collect data on previous SARS-CoV-2 infections, including PCR and serology testing. As such, immunity from previous infections might have affected the observed hazard ratios when comparing different variants. We had accurate history of vaccination and we controlled for this using both stratification (in the main analysis) and adjustment by regression in the comparison between Omicron sublineages. To be able to robustly take the potential confounding effect of previous infection into account, complete information for previous PCR-verified infections, serological and potentially antigen-based testing would be needed (and still one would miss infections not properly diagnosed). In the absence of all these, we opted to control in the regression models for calendar time as a proxy of any changes which occurred over time other than the circulating variant. Finally, because of the observational nature of our study, we cannot rule out that the differences in risk between variants may be biased by other residual or unmeasured confounding. This includes lack of data on sociodemographic factors and measured of level of deprivation (ethnicity, education level, income level).

### Conclusions

The unadjusted cumulative incidence of 28-day in-hospital mortality decreased throughout the study period, particularly during the Omicron period compared with preceding variant periods. However, when comparing the different variants, adjusted hazards of in-hospital mortality varied across different age groups, COVID-19 vaccination status, and different settings. Among participants ≥70 years, Alpha and Delta had an increased risk of in-hospital mortality vs. Omicron. This was not observed when restricting the analyses to unvaccinated participants, corroborating the protective and severity attenuating effects related to COVID-19 vaccination since vaccine uptake differed between the variant groups. Furthermore, in the Omicron group, BA.1 carried a higher risk of death than that seen with other more recently circulating BA.2 and BA.5 sublineages. Collectively, the herein observed decrease in in-hospital mortality seems to reflect a combined effect of immunity from vaccinations and previous infections, although differences in virulence between SARS-CoV-2 variants may also have contributed.

## Contributors

All authors were involved in conceptualization, data collection, investigation, methodology, resources. Funding was acquired by AS, FI, and MZ. FI and PH were responsible for the project administration. AS and PN supervised the project. IF designed and created the EuCARE database. PH curated and visualized the data and wrote the original draft. PH and ACL had full access to all the data in the study, performed the formal analyses, and validated the results. All authors contributed to reviewing and editing the manuscript.

## Data sharing statement

All code used in the study is available upon request by the corresponding author. The individual participant data underlying this article were subject to ethical approval and cannot be shared publicly. Data from the deidentified data sources are not freely available due to protection of the personal integrity of the participants.

## Ethics approval

The study was approved by Ethical Review Authorities for each centre. These were Comitato Etico Milano Area 1—Ospedale Luigi Sacco, Comissao de Etica para a Saude do CHLO—Hospital de Egas Moniz, Ethikkommission (EK) Medizinischen Fakultät der Heinrich-Heine-Universität Düsseldorf, Comité de Etica en investigacion del HRAE. “Dr Juan Graham Casasus”, HRA and Health and Care Research Wales (HCRW), The Swedish Ethical Review Authority, KEMRI Scientific and Ethics Review Unit (SERU), Komisji Bioetycznej Pomorskiego Uniwersytetu Medycznego w Szczecinie, Comitato Etico Indipendente—Fondazione PTV Policlinico Tor Vergata, and Vilniaus regioninio biomedicininiu tyrimu etikos komiteto.

## Declaration of interests

Anders Sönnerborg received grants for institutions from Gilead Sciences and GSK and consulting fees and speaker's honoraria from AstraZeneca, Moderna, GSK, Gilead Sciences, and MSD, all out of the present work. Björn-Erik Ole Jensen received consulting fees and speaker's honoraria from GSK, ViiV Healthcare, Gilead Sciences, MSD, Pfizer, AstraZeneca, Janssen-Cilag, Fresenius Medical Care and Falk Foundation, as well as support for attending meetings and travel from Gilead Sciences, all out of the present work. Milosz Parczewski received honoraria for presentation from Gilead Sciences, Abbvie, MSD, Janssen-Cilag, Roche, GSK, Virology Education, and Pfizer. Maurizio Zazzi received grants for institutions from Gilead Sciences, MSD, Theratechnologies and ViiV Healthcare and consulting fees and speaker's honoraria from GSK, Gilead Sciences, MSD and ViiV Healthcare, all out of the present work. All other authors reported no conflicts of interest.
