# Supplementary material for: In-hospital mortality during the wild-type, alpha, delta, and omicron SARS-CoV-2 waves: a multinational cohort study in the EuCARE project
Source: Lancet Reg Health Eur. 2024 Feb 2;38:100855. doi: 10.1016/j.lanepe.2024.100855 (PMC10928271; doi:10.1016/j.lanepe.2024.100855)
Supplement: Supplementary Material [file mmc1.docx]

Supplementary material for

**Twenty-Eight-Day In-Hospital Mortality During the Wild-type, Alpha, Delta, and Omicron SARS-CoV-2 Waves: A Multinational Cohort Study in the EuCARE Project**

**Authors:** Pontus Hedberg, Milosz Parczewski, Karol Serwin, Giulia Marchetti, Francesca Bai, Björn-Erik Ole Jensen, Joana P. V. Pereira, Francis Drobniewski, Henrik Reschreiter, Daniel Naumovas, Francesca Ceccherini-Silberstein, Gibran Horemheb Rubio Quintanares, Matilu Mwau, Cristina Toscano, Florian König, Nico Pfeifer, Maurizio Zazzi, Iuri Fanti, Francesca Incardona, Alessandro Cozzi-Lepri, Anders Sönnerborg, Pontus Nauclér

Correspondence to [pontus.hedberg@ki.se](mailto:pontus.hedberg@ki.se)

**Table of contents**

| **Content** | **Page** |
| --- | --- |
| Text S1. Descriptions of centres and their data sources | 4-7 |
| Figure S1. Study flow chart | 8 |
| Figure S2. National SARS-CoV-2 variant distribution from GISAID metadata | 9 |
| Figure S3. National SARS-CoV-2 Omicron sublineage distribution from GISAID metadata | 10 |
| Table S1. Characteristics of unvaccinated participants in the SARS-CoV-2 variant groups | 11-12 |
| Table S2. Characteristics of participants having received two or more COVID-19 vaccine doses in the Delta and Omicron groups | 13-15 |
| Table S3. Characteristics of participants in the Omicron BA.1, BA.2, and BA.5 sublineage groups | 16-18 |
| Figure S4. Cumulative incidences of 28-day in-hospital mortality among unvaccinated participants | 19 |
| Figure S5. Cumulative incidence and subdistribution hazard ratios for 28-day in-hospital mortality among participants having received two or more COVID-19 vaccine doses in the Delta and Omicron groups | 20 |
| Figure S6. Subdistribution hazard ratios for pairwise comparisons of 28-day in-hospital mortality when only including centres with >50 participants for each SARS-CoV-2-variant | 21 |
| Figure S7. Subdistribution hazard ratios for pairwise comparisons of 28-day in-hospital mortality when only including participants without a sequenced SARS-CoV-2 variant | 22 |
| Figure S8. Subdistribution hazard ratios for pairwise comparisons of 28-day in-hospital mortality when only including participants with a positive SARS-CoV-2 test from 14 days before to 1 day after the date of hospital admission | 23 |
| Figure S9. Subdistribution hazard ratios for pairwise comparisons of 28-day in-hospital mortality when only including participants from Sweden versus outside of Sweden | 24 |
| Figure S10. 28-day in-hospital mortality rates across age group, variant exposure group, and centre | 25 |
| Figure S11. Subdistribution hazard ratios for pairwise comparisons of 28-day in-hospital mortality in the variant groups using a >90% cut-off | 26 |
| Supplementary references | 27 |

**Text S1. Descriptions of centres and their data sources**

**ASST:**

Data from ASST Santi Paolo and Carlo included two main hospitals (San Paolo Hospital and San Carlo Hospital) in Milan, Italy. Demographic, clinical and microbiological data were collected from the electronic health record (EHR) system of ASST Santi Paolo and Carlo. Data about vaccination were collected from the Socio-health information system of the Lombardy region for residents in the Lombardy region and from the EHR system of ASST Santi Paolo and Carlo for participants residing in other regions. Participants who were discharged alive either could be discharged to their home or to another hospital or to subacute centres, rehabilitation centres or long-term facilities.

**CHLO:**

Data from CHLO included data from three hospitals (Hospital de Santa Cruz, Hospital Egas Moniz, Hospital São Francisco Xavier) included in the CHLO group in Lisbon, Portugal. The SClínico platform aggregates informational data pertaining the following: demographics, place of birth, migration, drug prescriptions, International Statistical Classification of Diseases and Related Health Problems 10th Revision (ICD-10) codes, all microbiological tests and all notified polymerase chain reaction (PCR) severe acute respiratory syndrome coronavirus 2 (SARS-CoV-2) positive test results performed in participants attending either of the 3 CHLO hospitals in Lisbon. The Electronic Health Registry (RSE) included information regarding coronavirus disease 2019 (COVID-19) vaccinations per participant, administered in Portugal. Participants who were discharged alive either could be discharged to their home or to another hospital or care facility not covered by the data sources used.

**HHU:**

Data from HHU included data from all wards of the Düsseldorf University hospital in Düsseldorf, Germany. Data was extracted from the electronic health records of the hospital. Some information was additionally extracted manually from the patient files. Participants who were discharged alive either could be discharged to their home or to another hospital or care facility not covered by our data sources.

**HRAE:**

Data from HRAE came from Hospital Regional de Alta Especialidad “Dr. Juan Graham Casasus” in Villahermosa, Mexico. Data was obtained both from the EHR system and from health records on paper. Microbiological data and vaccination data were retrieved from national epidemiological registries and local systems. Participants who were discharged alive were discharged to their home since no other hospitals or centres had the capacity to care for COVID-19 patients in the region.

**IMPERIAL:**

Data from IMPERIAL came from Poole Hospital in Poole, United Kingdom. All clinical, patient and laboratory data was originally extracted from the hospital electronic medical records and additional medical notes and entered into electronic case report forms (CRFs). Data was submitted electronically to the International Severe Acute Respiratory and emerging Infection Consortium (ISARIC) Study Center Oxford who coded the patient’s identity and extracted a coded data file covering the retrospective dates. Whole genome sequencing data was linked manually to this file by the PI and re-anonymised. Participants who were discharged alive could be sent to home or residential care centre from the hospital.

**KEMRI:**

All data collected by KEMRI came from one ward in Alupe Sub-County Hospital in Busia, Kenya. All data was extracted from paper-based in-patient medical files. Inpatient identification numbers as indicated in respective inpatient files were linked to the assigned site study identification number. Discharge represented either participant testing negative after hospitalisation or participant recommended for home-based care.

**KI:**

Data from KI came from six acute care hospitals (Danderyd Hospital, Karolinska University Hospital Huddinge, Karolinska University Hospital Solna, Norrtälje Hospital, Södersjukhuset, and Södertälje Hospital) in Stockholm County, Sweden. Data were linked using personal identification numbers, unique for each Swedish resident, from the TakeCare® Intelligence database, the Stockholm regional healthcare data warehouse (VAL), Statistics Sweden, SmiNet, the National Vaccination Register (NVR), and the Swedish Intensive Care Registry (SIR). The TakeCare® Intelligence database included all microbiological tests performed in patients attending the six acute care hospitals. VAL contains data from healthcare databases within the Stockholm Region, including demographics, migration, drug prescriptions, and International Statistical Classification of Diseases and Related Health Problems 10th Revision (ICD-10) codes from all inpatient stays and outpatient visits reimbursed by Region Stockholm [1]. Data from Statistics Sweden were used to collect information on region of birth and education level [2]. SmiNet contained all PCR SARS-CoV-2 positive test results notified in accordance with the Communicable Diseases Act [3]. The data from NVR included all COVID-19 vaccinations administered in Sweden to the Stockholm County population [4]. SIR, a national intensive care units (ICU) in Sweden, contained all ICU admissions in Stockholm County [5]. Participants who were discharged alive could either be discharged to their home or to another hospital or care facility not covered by the data sources used.

**PUM:**

Data from PUM came from PUM District Hospital in Szczecin, Poland. Data were extracted using personal identification number (unique for every Polish patient) and internal hospital number (also unique for every patient) from the electronic patient health records. These records hold both medical history, follow-up data as well as laboratory data integrated for every case. The database also includes basic laboratory and microbiologic data, admission and discharge, including initial and discharge diagnosis coded based on ICD-10 codes. Vaccination data were extracted from national database on COVID-19 vaccination and linked to patient data. Sequence data were linked from national COVID-19 sequencing program and local sequencing program, which were also linked to the patient data. Discharge from the hospital represented the final discharge for all hospital wards. This meant that participants discharged alive could be discharged to their home, a care facility or another hospital.

**UNITOV:**

All data from UNITOV came from Hospital Tor Vergata Roma in Rome, Italy. Demographics, epidemiological, clinical and laboratory data were retrieved by extracting them both from the EHR systems and from the paper medical records of the hospital where patients were followed, according to the European Regulation on the protection of personal data n. 679/2016 and the Italian Legislative Decree 196/2003. Discharge from the hospital represented the final discharge for all hospital wards. This meant that participants discharged alive could be discharged to their home, a care facility or another hospital.

**VULSK:**

Data from VULSK came from the VULSK hospital in Vilnius, Lithuania. All data was extracted from the hospital electronic medical records. Discharge from the hospital represented the final discharge for all hospital wards. This meant that participants discharged alive could be discharged to their home, a care facility or another hospital.

**Figure S1. Study flow chart**

**Note:** -

**Abbreviations:** COVID-19=Coronavirus disease 2019 **Figure S2. National SARS-CoV-2 variant distribution from GISAID metadata**

**Note:** The GISAID metadata was downloaded on 14 August 2023. Variants were classified based on the Variant column in the metadata. Samples who did not have any information in this column was classified as Others. Samples classified as Alpha with a collection date before 1 September 2020 were removed. This was also done for samples classified as Delta with a collection date before 1 October 2020.

**Abbreviations:** GISAID=Global Initiative on Sharing All Influenza Data, SARS-CoV-2=Severe acute respiratory syndrome coronavirus 2

**Figure S3. National SARS-CoV-2 Omicron sublineage distribution from GISAID metadata**

**Note:** The GISAID metadata was downloaded on 14 August 2023. Variants and subvariants were classified based on the Variant and Pango lineage columns in the metadata. No sequences were identified from Lithuania from 2 May 2022 to 10 October 2022, explaining the distribution of the BA.2 and BA.5 sublineages during that time period.

**Abbreviations:** GISAID=Global Initiative on Sharing All Influenza Data, SARS-CoV-2=Severe acute respiratory syndrome coronavirus 2

**Table S1. Characteristics of unvaccinated participants in the SARS-CoV-2 variant groups**

| **Variable** | **Overall (n=29,568)** | **Wild-type (n=19,524)** | **Alpha (n=5,718)** | **Delta (n=2,303)** | **Omicron (n=2,023)** |
| --- | --- | --- | --- | --- | --- |
| **Age, years** | 63.0 [49.0, 76.0] | 65.0 [52.0, 78.0] | 61.0 [49.0, 71.0] | 53.0 [40.0, 67.0] | 63.0 [40.0, 79.0] |
| 18 to 49 | 7,406 (25.0) | 4,250 (21.8) | 1,478 (25.8) | 996 (43.2) | 682 (33.7) |
| 50 to 69 | 11,057 (37.4) | 7,169 (36.7) | 2,579 (45.1) | 805 (35.0) | 504 (24.9) |
| 70 years or older | 11,105 (37.6) | 8,105 (41.5) | 1,661 (29.0) | 502 (21.8) | 837 (41.4) |
| **Male sex** | 17,071 (57.7) | 11,631 (59.6) | 3,333 (58.3) | 1,225 (53.2) | 882 (43.6) |
| **Comorbidities** |  |  |  |  |  |
| Cancer | 1,852 (6.3) | 1,351 (6.9) | 250 (4.4) | 80 (3.5) | 171 (8.5) |
| Cardiac or cerebrovascular disease | 6,695 (22.6) | 4,985 (25.5) | 889 (15.5) | 328 (14.2) | 493 (24.4) |
| Chronic kidney disease | 2,679 (9.1) | 1,984 (10.2) | 304 (5.3) | 178 (7.7) | 213 (10.5) |
| Chronic liver disease | 668 (2.3) | 456 (2.3) | 102 (1.8) | 51 (2.2) | 59 (2.9) |
| Chronic lung disease | 3,927 (13.3) | 2,750 (14.1) | 651 (11.4) | 225 (9.8) | 301 (14.9) |
| Diabetes | 5,830 (19.7) | 4,214 (21.6) | 868 (15.2) | 348 (15.1) | 400 (19.8) |
| Hypertension | 10,976 (37.1) | 7,839 (40.2) | 1,812 (31.7) | 581 (25.2) | 744 (36.8) |
| Immunocompromised | 1,945 (6.6) | 1,395 (7.1) | 311 (5.4) | 82 (3.6) | 157 (7.8) |
| Neurologic conditions | 1,946 (6.6) | 1,471 (7.5) | 170 (3.0) | 105 (4.6) | 200 (9.9) |
| Obesity | 5,485 (18.6) | 3,614 (18.5) | 1,190 (20.8) | 340 (14.8) | 341 (16.9) |
| Number of comorbidities | 1.0 [0.0, 2.0] | 1.0 [0.0, 2.0] | 1.0 [0.0, 2.0] | 1.0 [0.0, 2.0] | 1.0 [0.0, 2.0] |
| 0 | 10,351 (35.0) | 6,210 (31.8) | 2,385 (41.7) | 1,093 (47.5) | 663 (32.8) |
| 1 | 7,295 (24.7) | 4,729 (24.2) | 1,508 (26.4) | 583 (25.3) | 475 (23.5) |
| 2 | 5,378 (18.2) | 3,754 (19.2) | 920 (16.1) | 318 (13.8) | 386 (19.1) |
| 3 | 3,605 (12.2) | 2,582 (13.2) | 554 (9.7) | 194 (8.4) | 275 (13.6) |
| 4 or more | 2,939 (9.9) | 2,249 (11.5) | 351 (6.1) | 115 (5.0) | 224 (11.1) |
| **Admission vitals and lab values** |  |  |  |  |  |
| Respiratory rate, breaths/minute | 24.0 [20.0, 30.0] | 24.0 [20.0, 30.0] | 24.0 [21.0, 30.0] | 22.0 [18.0, 28.0] | 22.0 [19.0, 28.0] |
| Missing data | 9,999 (33.8) | 6,444 (33.0) | 1,991 (34.8) | 839 (36.4) | 725 (35.8) |
| Peripheral oxygen saturation, % | 92.0 [88.0, 95.0] | 92.0 [88.0, 95.0] | 92.0 [89.0, 95.0] | 94.0 [90.0, 96.0] | 94.0 [90.0, 96.0] |
| Missing data | 9,131 (30.9) | 5,588 (28.6) | 1,984 (34.7) | 827 (35.9) | 732 (36.2) |
| C-reactive protein, mg/L | 61.3 [19.0, 125.0] | 64.0 [21.0, 127.9] | 70.0 [27.5, 130.0] | 36.5 [7.9, 99.0] | 28.0 [8.0, 89.4] |
| Missing data | 3,239 (11.0) | 1,817 (9.3) | 532 (9.3) | 246 (10.7) | 644 (31.8) |
| White blood cell count, 10^9^ cells/L | 6.8 [5.0, 9.4] | 7.0 [5.2, 9.6] | 6.3 [4.7, 8.4] | 6.4 [4.7, 8.7] | 7.7 [5.5, 10.8] |
| Missing data | 2,944 (10.0) | 1,774 (9.1) | 493 (8.6) | 206 (8.9) | 471 (23.3) |
| Platelet count, 10^9^ cells/L | 201.0 [156.8, 261.0] | 204.0 [159.0, 263.0] | 196.0 [154.0, 253.0] | 192.0 [148.0, 249.0] | 203.0 [157.0, 270.0] |
| Missing data | 3,996 (13.5) | 2,224 (11.4) | 715 (12.5) | 542 (23.5) | 515 (25.5) |
| Lymphocyte count, 10^9^ cells/L | 1.0 [0.7, 1.4] | 1.0 [0.7, 1.4] | 1.0 [0.7, 1.3] | 1.0 [0.7, 1.4] | 1.1 [0.7, 1.6] |
| Missing data | 7,564 (25.6) | 4,956 (25.4) | 1,097 (19.2) | 423 (18.4) | 1,088 (53.8) |

**Note:** When comparing the four groups, *P* values from Kruskal-Wallis tests and Chi squared tests were <0.001 for all variables except chronic liver disease (*P*=0.016)

**Abbreviations:** SARS-CoV-2=Severe acute respiratory syndrome coronavirus 2

**Table S2. Characteristics of participants having received two or more COVID-19 vaccine doses in the Delta and Omicron groups**

| **Variable** | **Overall (n=7,377)** | **Delta (n=985)** | **Omicron (n=6,392)** |
| --- | --- | --- | --- |
| **Age, years** | 74.0 [57.0, 83.0] | 74.0 [62.0, 83.0] | 74.0 [57.0, 83.0] |
| 18 to 49 | 1,313 (17.8) | 118 (12.0) | 1,195 (18.7) |
| 50 to 69 | 1,723 (23.4) | 270 (27.4) | 1,453 (22.7) |
| 70 years or older | 4,341 (58.8) | 597 (60.6) | 3,744 (58.6) |
| **Male sex** | 3,907 (53.0) | 557 (56.5) | 3,350 (52.4) |
| **Comorbidities** |  |  |  |
| Cancer | 1,097 (14.9) | 139 (14.1) | 958 (15.0) |
| Cardiac or cerebrovascular disease | 2,939 (39.8) | 391 (39.7) | 2,548 (39.9) |
| Chronic kidney disease | 1,477 (20.0) | 214 (21.7) | 1,263 (19.8) |
| Chronic liver disease | 261 (3.5) | 37 (3.8) | 224 (3.5) |
| Chronic lung disease | 1,492 (20.2) | 212 (21.5) | 1,280 (20.0) |
| Diabetes | 1,706 (23.1) | 257 (26.1) | 1,449 (22.7) |
| Hypertension | 4,141 (56.1) | 557 (56.5) | 3,584 (56.1) |
| Immunocompromised | 1,538 (20.8) | 178 (18.1) | 1,360 (21.3) |
| Neurologic conditions | 1,034 (14.0) | 141 (14.3) | 893 (14.0) |
| Obesity | 1,159 (15.7) | 171 (17.4) | 988 (15.5) |
| Number of comorbidities | 2.0 [1.0, 3.0] | 2.0 [1.0, 3.0] | 2.0 [1.0, 3.0] |
| 0 | 1,165 (15.8) | 122 (12.4) | 1,043 (16.3) |
| 1 | 1,404 (19.0) | 179 (18.2) | 1,225 (19.2) |
| 2 | 1,630 (22.1) | 255 (25.9) | 1,375 (21.5) |
| 3 | 1,459 (19.8) | 219 (22.2) | 1,240 (19.4) |
| 4 or more | 1,719 (23.3) | 210 (21.3) | 1,509 (23.6) |
| **COVID-19 vaccine doses ^a^** |  |  |  |
| 2 doses | 2,702 (36.6) | 903 (91.7) | 1,799 (28.1) |
| 3 doses or more | 4,675 (63.4) | 82 (8.3) | 4,593 (71.9) |
| **Admission vitals and lab values** |  |  |  |
| Respiratory rate, breaths/minute | 22.0 [20.0, 27.0] | 24.0 [20.0, 30.0] | 22.0 [20.0, 26.0] |
| Missing data | 1,565 (21.2) | 332 (33.7) | 1,233 (19.3) |
| Peripheral oxygen saturation, % | 94.0 [91.0, 96.0] | 93.0 [89.0, 95.0] | 94.0 [91.0, 96.0] |
| Missing data | 1,589 (21.5) | 338 (34.3) | 1,251 (19.6) |
| C-reactive protein, mg/L | 33.0 [7.0, 95.0] | 47.4 [9.5, 112.0] | 31.0 [7.0, 92.0] |
| Missing data | 722 (9.8) | 124 (12.6) | 598 (9.4) |
| White blood cell count, 10^9^ cells/L | 8.4 [6.3, 11.2] | 7.8 [5.7, 9.9] | 8.5 [6.4, 11.4] |
| Missing data | 573 (7.8) | 112 (11.4) | 461 (7.2) |
| Platelet count, 10^9^ cells/L | 206.0 [158.0, 270.0] | 193.0 [144.2, 250.8] | 208.0 [160.0, 271.0] |
| Missing data | 620 (8.4) | 123 (12.5) | 497 (7.8) |
| Lymphocyte count, 10^9^ cells/L | 1.1 [0.7, 1.6] | 1.0 [0.6, 1.5] | 1.1 [0.7, 1.6] |
| Missing data | 4,333 (58.7) | 341 (34.6) | 3,992 (62.5) |

**Note:** When comparing the four groups, *P* values from Kruskal-Wallis tests and Chi squared tests were <0.001 for all variables except age (*P*=0.196), lymphocyte count (*P*=0.003), sex (*P*=0.017), cancer (*P*=0.502), cardiac or cerebrovascular disease (*P*=0.949), chronic kidney disease (*P*=0.164), chronic liver disease (*P*=0.760), chronic lung disease (*P*=0.295), diabetes (*P*=0.020), hypertension (*P*=0.805), immunocompromised (*P*=0.024), neurologic conditions (*P*=0.810), and obesity (*P*=0.139).

**Abbreviations:** COVID-19=Coronavirus disease 2019

a. 709 participants (2%) were excluded from the analyses due to unknown vaccination status before the hospitalisation

**Table S3. Characteristics of participants in the Omicron BA.1, BA.2, and BA.5 sublineage groups**

| **Variable** | **Overall (n=6,274)** | **BA.1 (n=1,572)** | **BA.2 (n=2,399)** | **BA.5 (n=2,303)** |
| --- | --- | --- | --- | --- |
| **Age, years** | 73.0 [56.0, 83.0] | 67.0 [44.0, 80.0] | 73.0 [57.0, 83.0] | 76.0 [62.0, 83.5] |
| 18 to 49 | 1,226 (19.5) | 463 (29.5) | 422 (17.6) | 341 (14.8) |
| 50 to 69 | 1,457 (23.2) | 387 (24.6) | 587 (24.5) | 483 (21.0) |
| 70 years or older | 3,591 (57.2) | 722 (45.9) | 1,390 (57.9) | 1,479 (64.2) |
| **Male sex** | 3,271 (52.1) | 756 (48.1) | 1,278 (53.3) | 1,237 (53.7) |
| **Comorbidities** |  |  |  |  |
| Cancer | 912 (14.5) | 193 (12.3) | 357 (14.9) | 362 (15.7) |
| Cardiac or cerebrovascular disease | 2,363 (37.7) | 461 (29.3) | 937 (39.1) | 965 (41.9) |
| Chronic kidney disease | 1,176 (18.7) | 265 (16.9) | 498 (20.8) | 413 (17.9) |
| Chronic liver disease | 219 (3.5) | 53 (3.4) | 91 (3.8) | 75 (3.3) |
| Chronic lung disease | 1,188 (18.9) | 261 (16.6) | 477 (19.9) | 450 (19.5) |
| Diabetes | 1,374 (21.9) | 292 (18.6) | 515 (21.5) | 567 (24.6) |
| Hypertension | 3,348 (53.4) | 718 (45.7) | 1,284 (53.5) | 1,346 (58.4) |
| Immunocompromised | 1,157 (18.4) | 249 (15.8) | 506 (21.1) | 402 (17.5) |
| Neurologic conditions | 868 (13.8) | 223 (14.2) | 342 (14.3) | 303 (13.2) |
| Obesity | 932 (14.9) | 212 (13.5) | 340 (14.2) | 380 (16.5) |
| Number of comorbidities | 2.0 [1.0, 3.0] | 2.0 [1.0, 3.0] | 2.0 [1.0, 3.0] | 2.0 [1.0, 3.0] |
| 0 | 1,120 (17.9) | 390 (24.8) | 404 (16.8) | 326 (14.2) |
| 1 | 1,290 (20.6) | 341 (21.7) | 476 (19.8) | 473 (20.5) |
| 2 | 1,383 (22.0) | 349 (22.2) | 516 (21.5) | 518 (22.5) |
| 3 | 1,149 (18.3) | 224 (14.2) | 466 (19.4) | 459 (19.9) |
| 4 or more | 1,332 (21.2) | 268 (17.0) | 537 (22.4) | 527 (22.9) |
| **COVID-19 vaccine doses ^a^** |  |  |  |  |
| Unvaccinated | 1,359 (22.1) | 630 (41.1) | 347 (15.0) | 382 (16.7) |
| 1 dose | 136 (2.2) | 48 (3.1) | 52 (2.2) | 36 (1.6) |
| 2 doses | 1,014 (16.5) | 402 (26.2) | 383 (16.5) | 229 (10.0) |
| 3 doses or more | 3,629 (59.1) | 454 (29.6) | 1,536 (66.3) | 1,639 (71.7) |
| **Admission vitals and lab values** |  |  |  |  |
| Respiratory rate, breaths/minute | 22.0 [20.0, 28.0] | 24.0 [20.0, 42.5] | 22.0 [20.0, 26.0] | 22.0 [20.0, 26.0] |
| Missing data | 1,662 (26.5) | 772 (49.1) | 634 (26.4) | 256 (11.1) |
| Peripheral oxygen saturation, % | 94.0 [91.0, 96.0] | 93.0 [88.0, 96.0] | 94.0 [91.0, 96.0] | 94.0 [91.0, 96.0] |
| Missing data | 1,646 (26.2) | 848 (53.9) | 669 (27.9) | 129 (5.6) |
| C-reactive protein, mg/L | 28.5 [6.4, 88.0] | 18.0 [5.0, 68.0] | 23.0 [5.0, 82.1] | 39.0 [10.0, 105.0] |
| Missing data | 881 (14.0) | 414 (26.3) | 223 (9.3) | 244 (10.6) |
| White blood cell count, 10^9^ cells/L | 8.4 [6.2, 11.3] | 7.7 [5.5, 10.1] | 8.6 [6.5, 11.5] | 8.6 [6.3, 11.8] |
| Missing data | 638 (10.2) | 372 (23.7) | 178 (7.4) | 88 (3.8) |
| Platelet count, 10^9^ cells/L | 206.0 [158.0, 270.0] | 197.0 [152.2, 252.0] | 209.0 [159.0, 272.0] | 209.0 [159.0, 278.0] |
| Missing data | 693 (11.0) | 386 (24.6) | 206 (8.6) | 101 (4.4) |
| Lymphocyte count, 10^9^ cells/L | 1.1 [0.7, 1.6] | 1.0 [0.6, 1.6] | 1.2 [0.8, 1.7] | 1.0 [0.7, 1.6] |
| Missing data | 3,710 (59.1) | 787 (50.1) | 1,453 (60.6) | 1,470 (63.8) |

**Note:** When comparing the four groups, *P* values from Kruskal-Wallis tests and Chi squared tests were <0.001 for all variables except cancer (*P*=0.010), chronic kidney disease (*P*=0.004), chronic liver disease (*P*=0.579), chronic lung disease (*P*=0.023), neurologic conditions (*P*=0.495), and obesity (*P*=0.017).

**Abbreviations:** COVID-19=Coronavirus disease 2019

a. 136 participants (2%) were excluded from the analyses due to unknown vaccination status before the hospitalisation

**Figure S4. Cumulative incidences of 28-day in-hospital mortality among unvaccinated participants**

**
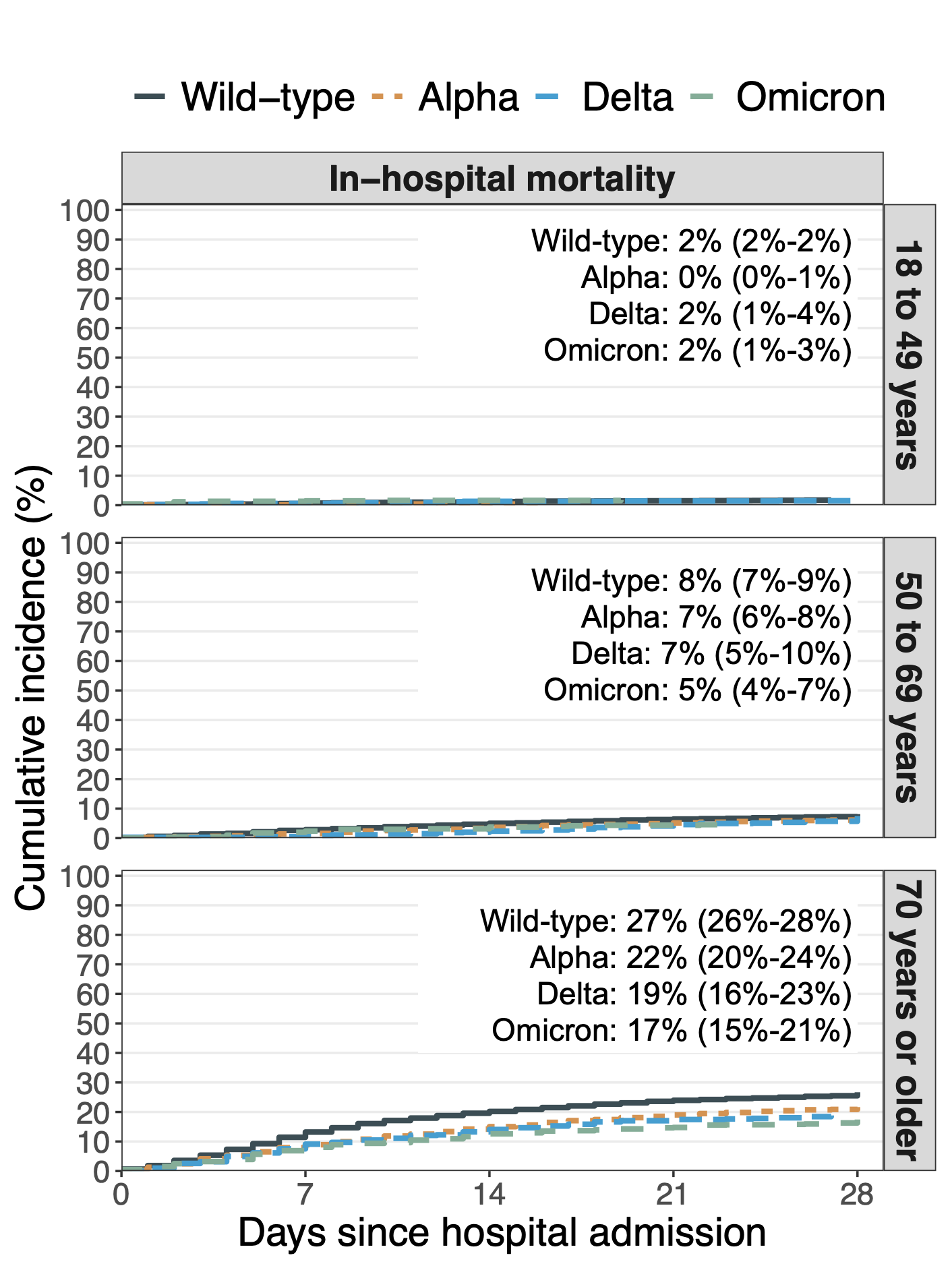
**

**Note:** The text in the figure describes the cumulative incidence (95% confidence interval) at 28 days except for Wild-type 18 to 49 years (day 27), Alpha 18 to 49 years (day 16), and Omicron 18 to 49 years (day 19).

**Figure S5. Cumulative incidence and subdistribution hazard ratios for 28-day in-hospital mortality among participants having received two or more COVID-19 vaccine doses in the Delta and Omicron groups**

**
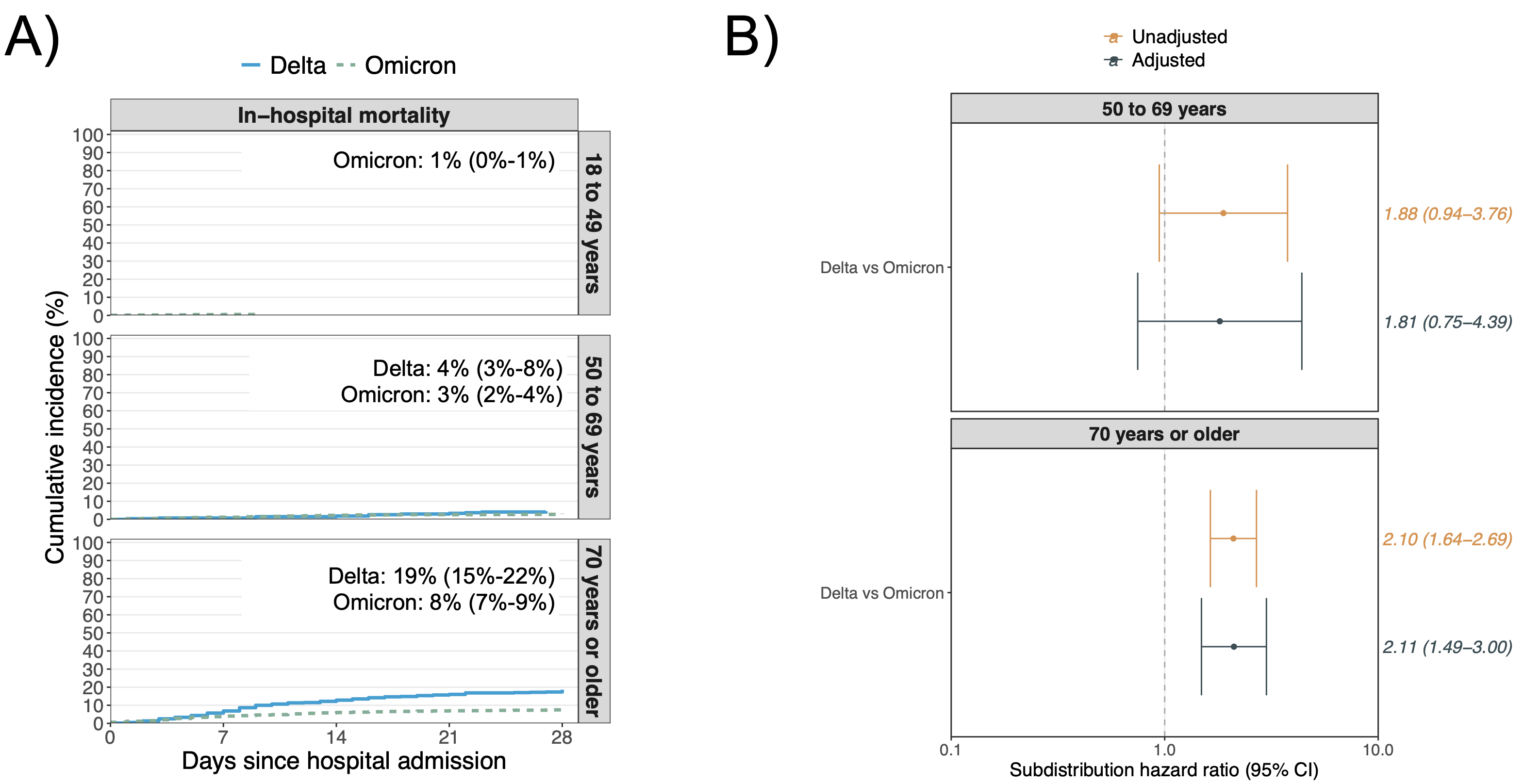
**

**Note:** The text in the figure describes the cumulative incidence at 28 days except for Delta 50 to 69 years (day 27), and Omicron 18 to 49 years (day 9). The adjusted models were adjusted for age, sex, calendar time, and all studied comorbidities. Only one knot set at 9 March 2022 was used for this analysis. Centre was included as a stratification factor in all models.

**Abbreviations:** CI=Confidence interval

**Figure S6. Subdistribution hazard ratios for pairwise comparisons of 28-day in-hospital mortality when only including centres with >50 participants for each SARS-CoV-2-variant**

**Note:** The adjusted models were adjusted for age, sex, calendar time, and all studied comorbidities. Centre was included as a stratification factor in all models.

**Abbreviations:** CI=Confidence interval

**Figure S7. Subdistribution hazard ratios for pairwise comparisons of 28-day in-hospital mortality when only including participants without a sequenced SARS-CoV-2 variant**

**Note:** The adjusted models were adjusted for age, sex, calendar time, and all studied comorbidities. Centre was included as a stratification factor in all models.

**Abbreviations:** CI=Confidence interval

**Figure S8. Subdistribution hazard ratios for pairwise comparisons of 28-day in-hospital mortality when only including participants with a positive SARS-CoV-2 test from 14 days before to 1 day after the date of hospital admission**

**Note:** The adjusted models were adjusted for age, sex, calendar time, and all studied comorbidities. Centre was included as a stratification factor in all models.

**Abbreviations:** CI=Confidence interval

**Figure S9. Subdistribution hazard ratios for pairwise comparisons of 28-day in-hospital mortality when only including participants from Sweden versus outside of Sweden**

**
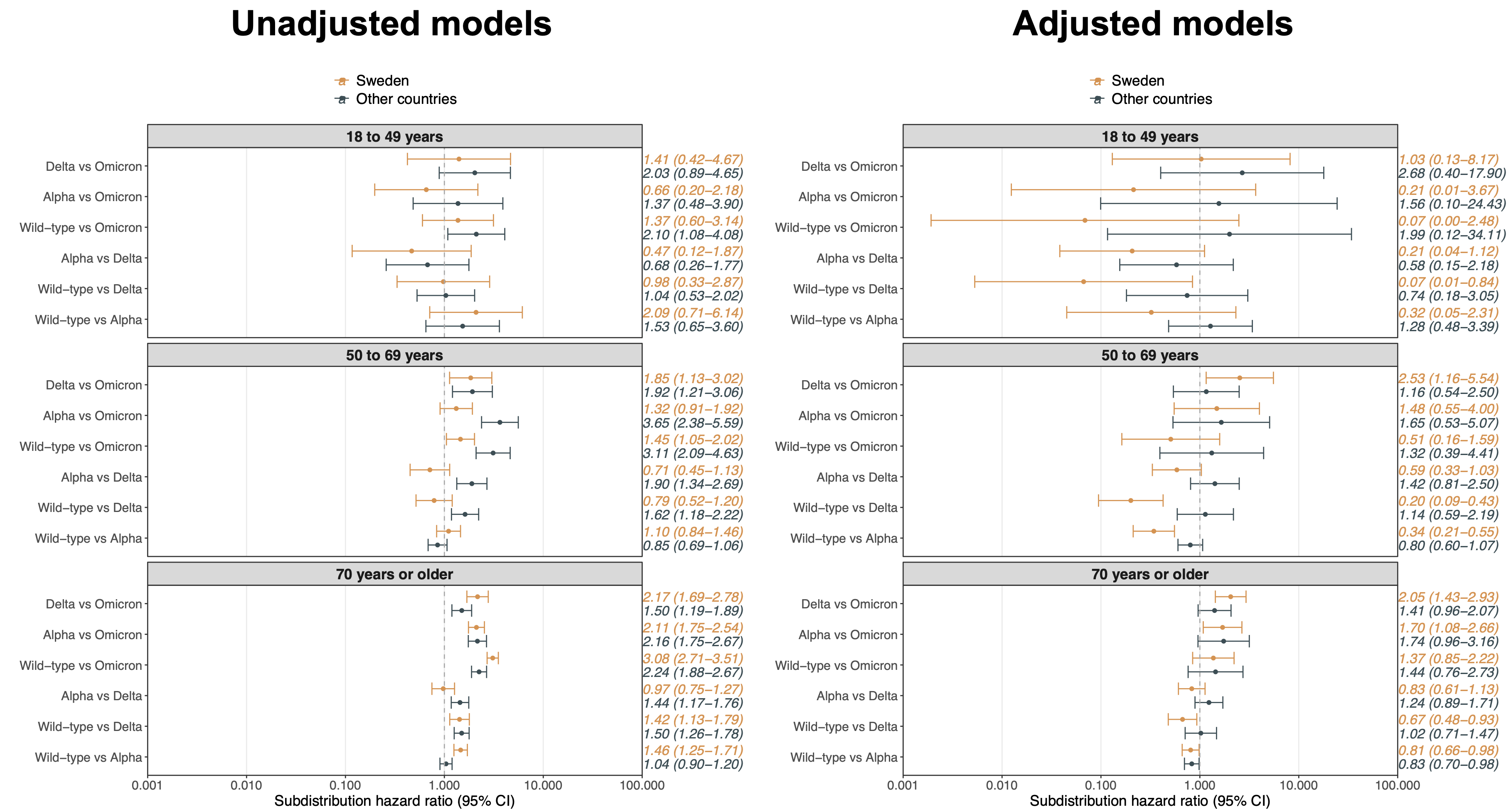
**

**Note:** The adjusted models were adjusted for age, sex, calendar time, and all studied comorbidities. Centre was included as a stratification factor in the models for participants outside of Sweden.

**Abbreviations:** CI=Confidence interval

**Figure S10. 28-day in-hospital mortality rates across age group, variant exposure group, and centre**

**Note:** The values inside the parentheses represent the number of participants dying / the total number of participants.

**Abbreviations:** SARS-CoV-2=Severe acute respiratory syndrome coronavirus 2

**Figure S11. Subdistribution hazard ratios for pairwise comparisons of 28-day in-hospital mortality in the variant groups using a >90% cut-off**

**Note:** The adjusted models were adjusted for age, sex, calendar time, and all studied comorbidities. Centre was included as a stratification factor in all models.

**Abbreviations:** CI=Confidence interval

**Supplementary references**

1. Hedberg P, Granath F, Bruchfeld J, *et al.* Post COVID-19 condition diagnosis: A population-based cohort study of occurrence, associated factors, and healthcare use by severity of acute infection. 2022.

2. Drefahl S, Wallace M, Mussino E, *et al.* A population-based cohort study of socio-demographic risk factors for COVID-19 deaths in Sweden. *Nat Commun* 2020; **11**: 5097.

3. Rolfhamre P, Janson A, Arneborn M, Ekdahl K. SmiNet-2: Description of an internet-based surveillance system for communicable diseases in Sweden. *Euro Surveill Bull Eur Sur Mal Transm Eur Commun Dis Bull* 2006; **11**: 15—16.

4. Hedberg P, Sotoodeh A, Askling HH, Nauclér P. Sociodemographic disparities affect COVID‐19 vaccine uptake in non‐elderly adults with increased risk of severe COVID‐19. *J Intern Med* 2023: joim.13700.

5. Zettersten E, Engerström L, Bell M, *et al.* Long-term outcome after intensive care for COVID-19: differences between men and women—a nationwide cohort study. *Crit Care* 2021; **25**: 86.
